# Prognostic value of epicardial adipose tissue in heart failure: a systematic review and meta-analysis

**DOI:** 10.3389/fcvm.2025.1618614

**Published:** 2025-06-26

**Authors:** Qingqun Wu, Junhan Guo, Fengru Liu, Chenchen Dong, Yu Wang, Lu Liu, Shulong Zhang

**Affiliations:** Heart Center, Affiliated Zhongshan Hospital of Dalian University, Dalian, China

**Keywords:** epicardial adipose tissue, heart failure, prognosis, prediction, ejection fraction

## Abstract

**Background:**

Epicardial adipose tissue (EAT) essentially affects the pathophysiologic development of heart failure (HF), while existing research has not well elucidated its prognostic value for outcome. The present study aims at including relevant studies for systematically assessing its prognostic value in HF patients.

**Methods:**

The studies assessing the way EAT was employed to predict adverse HF outcomes were included from PubMed, Web of Science, Embase, and the Cochrane Library databases, and relevant data were accurately extracted. The primary outcome included the composite outcome of HF hospitalization and all-cause mortality (ACM). Secondary outcomes were the composite outcome of cardiovascular death and HF hospitalization and HF rehospitalization. We combined the standard mean difference (SMD) of EAT in HF patients with and without adverse events (AEs) and the EAT to adverse outcome hazard ratio (HR).

**Results:**

The nine included studies involved 1,939 HF patients and 329 control populations. HF patients with AEs presented a higher EAT vs. those without (SMD: 3.33, CI: 0.96–5.69, *p* = 0.006, *I*^2^ = 98%). Increased EAT per unit indicated a higher risk of the composite outcome of HF hospitalization and all-cause mortality (HR: 1.28, CI: 1.42–1.85, *p* = 0.0002, *I*^2^ = 93%) and HF readmission (HR: 1.05, CI: 1.03–1.07, *p* < 0.001, *I*^2^ = 10%), but did not relate to that of cardiovascular death and HF hospitalization (HR: 1.17, CI: 0.99–1.39, *p* = 0.06, *I*^2^ = 76%). The pooled AUC value for EAT to predict the primary outcome was 0.74 (CI: 0.66–0.82, *p* = 0.018, *I*^2^ = 70.1%) in HF patients with EF >40%.

**Conclusion:**

EAT is considered a clinical predictor of the composite outcome of HF hospitalization and ACM and may contribute to the prediction of poor prognosis in HF patients.

**Systematic Review Registration:**

https://www.crd.york.ac.uk/, identifier [CRD420250653252].

## 1 Introduction

Epicardial adipose tissue (EAT) lies between the myocardium and the epicardium, and is a type of adipose tissue with endocrine and systemic metabolic regulatory functions (1). EAT not only provides mechanical protection for arteries and nerves and energy supply to the myocardium, but also buffers against fatty acid toxicity levels (2). However, because there is no physical barrier separating EAT from the myocardial surface and it shares the same microcirculation, adipose tissue can easily penetrate the myocardium and interact directly with it as EAT expands and infiltrates. EAT secretes large amounts of adipokines and active products such as adrenomedullin and lipocalin (3, 4), leading to myocardial lipotoxicity, increased myocardial fibrosis, insulin resistance, and pathologically pro-inflammatory transformations (5–8), thus participating in heart failure (HF) process.

The pathophysiological mechanism pertaining to EAT in HF with reduced ejection fraction (HFrEF) vs. preserved ejection fraction (HFpEF) is still being studied. But numerous studies have shown that EAT mass or volume indices are lower in HFrEF than healthy controls, while EAT mass is higher in HFpEF (9–12). And patient EAT is associated with impaired exercise capacity (13) and left ventricular dysfunction (10–12, 14). In one study, increased EAT in HFrEF was associated with a lower risk of adverse events (AEs), whereas increased EAT in HFpEF was associated with a higher risk (13). However, it remains controversial whether EAT itself is associated with adverse outcomes in different types of HF, or whether these associations show opposite trends. In addition, differences in EAT measurements protocols, as well as EAT metrics and their thresholds for heterogeneity, are not yet clear.

Therefore, the study aimed at evaluating whether EAT volume or thickness, as an imaging biomarker, can predict poor prognosis in patients with different types of HF and to provide comprehensive data on the heterogeneity of EAT measurement protocols and metrics originating from different studies.

## 2 Research methods

This study was reported following the Preferred Reporting Items for Systematic Reviews and Meta-Analyses (PRISMA) guidelines statement (15). The study protocol has been registered in PROSPERO (registry No.: CRD420250653252).

### 2.1 Search strategy

PubMed, Web of science, Embase, and the Cochrane Library databases were subjected to a systematic search by two authors (QQ.W. and JH.G.) from their inception to January 2, 2025. Search terms mainly included “epicardial adipose tissue” “heart failure” “prognosis”. In addition, references to all included studies and published systematic reviews and meta-analyses were also screened individually to confirm other studies that met the criteria.

### 2.2 Exclusion criteria

Two authors (QQ.W. and JH.G.) independently screened the literature records to determine if there were potentially eligible articles. A study was eligible if: (i) patients were diagnosed with HF; (ii) the study was to evaluate the prognostic value of EAT in heart failure; (iii) EAT thickness, mass, or volume measured by coronary computed tomography angiography (CCTA), cardiac magnetic resonence (CMR), or echocardiography was reported; (iv) at least one outcome of interest was reported. A study was not eligible if met any of the five criteria: (i) conference abstracts, letters, or reviews; (ii) failure to report EAT thickness, mass, or volume; (iii) lack of desired outcome; (iv) failure to provide EAT thickness, mass, or volume to hazard ratio (HR) of adverse outcomes; (v) non-English literature. In case of disagreement, the third author (FR.L.) made the final decision.

### 2.3 Data extraction

Two authors (QQ.W. and JH.G.) independently extracted patient baseline features and data results according to predefined criteria. Data collected included: first author, study type, year of publication, country, study population, body mass index (BMI), age, gender, follow-up duration, left ventricular ejection fraction (LVEF), ischemic cause, atrial fibrillation, EAT measurements, and EAT assays. In addition, we extracted the HR values of EAT measurements to adverse outcomes and the area under the receiver operating characteristic curve (ROC-AUC) for predicting main outcomes in the included studies. Any disagreements encountered were agreed upon through consultation with the third author (FR.L.).

### 2.4 Outcome and subgroup analysis

The primary outcome referred to the composite outcome of HF hospitalization and all-cause mortality (ACM). Secondary outcomes included the composite outcome of cardiovascular death and HF hospitalization and HF rehospitalization. Predefined subgroup analyses were based on (1) LVEF levels, (2) age, (3) EAT indicator, and (4) country of origin.

### 2.5 Quality assessment

This study assessed the quality of the included studies by using the Newcastle-Ottawa Scale (NOS). The NOS scale evaluates each study from three aspects: population selection, comparability, and research results, and contains a total of 8 items and 9 scoring items. the quality of the studies depends on the score. 7–9 indicated high-quality literature.

### 2.6 Statistical analysis

For continuous variables, pooled standard mean differences (SMDs) and 95% CIs were obtained by separately calculating and comparing SMDs for EAT in patients with and without events. The prognostic value of EAT for primary and secondary outcomes was assessed based on the combined HR and 95% CI. Heterogeneity was assessed using the Cochrane Q-test and the *I*^2^ statistic; and *P*-values <0.10 and *I*^2^ values >25% were considered significantly heterogeneous, and random-effects models served for synthesizing effect sizes; conversely, fixed-effects models were used. Sensitivity analyses were performed using a single-study exclusion approach, thereby evaluating the impact of each study on combined results and the result robustness. Three methods, including Funnel plots, Egger tests and Begg's tests, engaged in assessing publication bias. To further assess how publication bias affected pooled results, we used the trim and fill method for the analysis of theoretical missing studies. Statistical analysis relied on RevMan 5.4 and Stata 18.0, and a two-tailed *p* < 0.05 reported statistical significance.

## 3 Research results

### 3.1 Study selection and quality assessment

**Figure 1:**
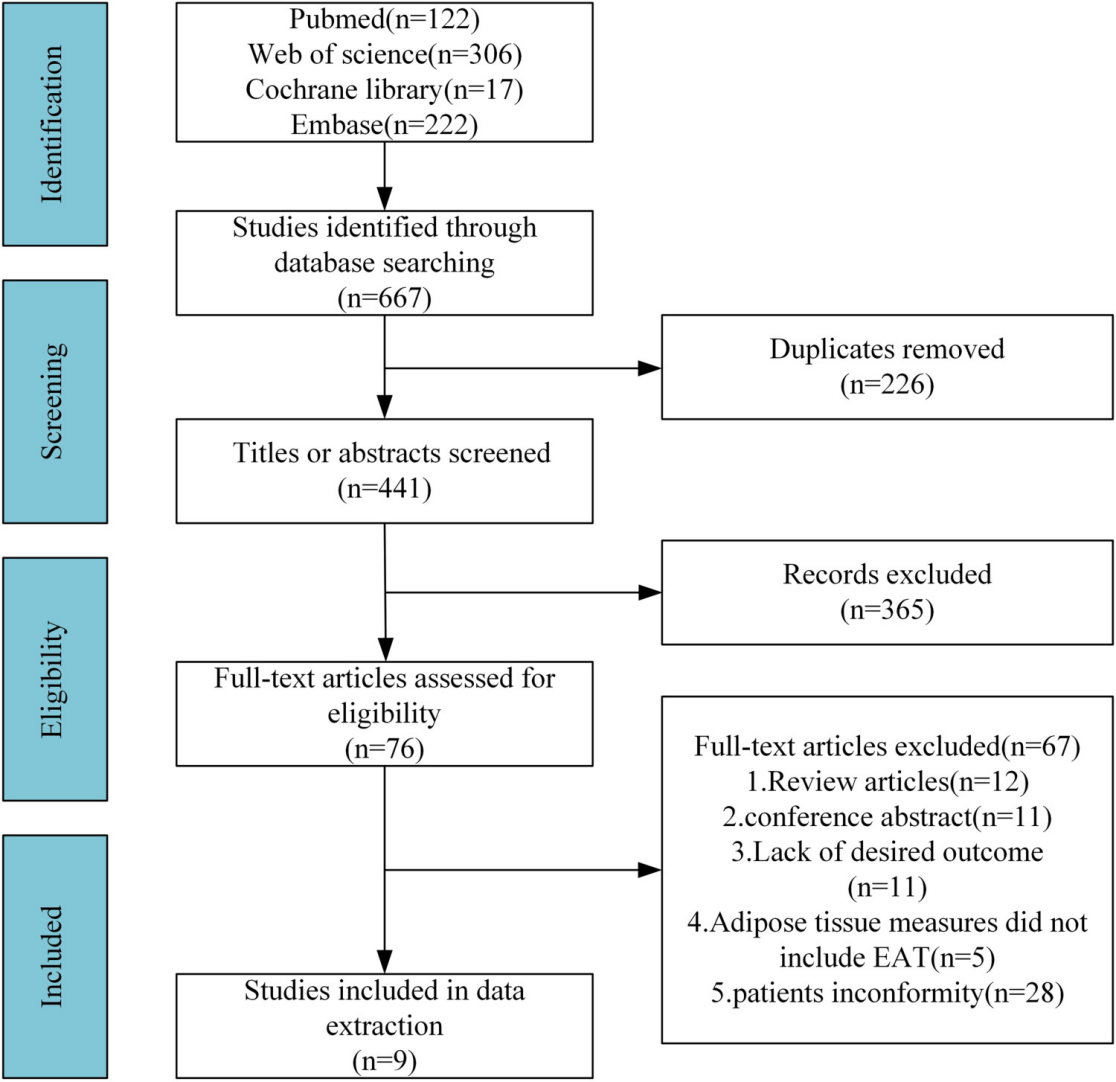
Study screening and selection flowchart.

Of the initial 667 papers retrieved, 441 papers remained after eliminating duplicates. After screening titles and abstracts, we obtained 76 potentially eligible studies. After full-text review, 67 studies that failed in satisfying the inclusion criteria were excluded. Hence, we included the remaining 9 studies in the final analysis, including 1,939 patients with HF and 329 control populations (13, 16–23) (Figure 1).

The included studies were subjected to quality assessment by virtue of the Newcastle-Ottawa scale, finding that all nine studies were of high quality with scores of above 7 in our meta-analysis (Supplementary Table S1).

### 3.2 Patient characteristics

According to Table 1 that lists the basic characteristics of the nine cohort studies, five were single- or multi-center prospective cohort studies. For the patient population, HFrEF and mid-range ejection fraction (HFmrEF) were reported in two studies, HFpEF was reported in seven studies, and the type of HF reported in one study was unclear. The median age ranged from 57 to 73 years, with 56.4% being male. The median follow-up ranged from 6 to 49.8 months. For EAT measurements, two studies reported EAT thickness using echocardiography, two studies reported EAT volume using CCTA, and another five studies measured EAT volume by CMR. HFpEF patients had remarkably higher EAT thickness vs. HFrEF patients, and EAT volume or thickness was higher in HFpEF patients and lower in HFrEF patients vs. controls.

**Table 1:** Summary of characteristics of all included studies.

Author, year	Country	Study design	Study population	LVEF (%)	Age (year)	BMI (kg/m^2^)	Sex (male, %)	Ischemic cause	AF	Follow time	EAT measurement	EAT thickness/mass/volume
Parisi, 2020 (18)	Italy	Cohort Pro	HFrEF: 69	HFrEF: 35.7 ± 7.8	HFrEF: 64.5 ± 10	NA	HFrEF: 61 (88.4)	HFrEF: 47 (68.1)	NA	49.8 months	Echocardiogram: EAT thickness	HFrEF: 10.2 ± 3.6 mm
Pugliese, 2021 (14)	Italy	Cohort Pro	HFrEF: 205 HFpEF:188 Control:44	NA	HFrEF: 65 (55–74) HFpEF: 73 (64–80) Control: 61 (54–70)	HFrEF: 27.4 (21.2–32.8) HFpEF: 31.5 (28.6–35.9) Control: 22.6 (21.8–24.2)	HFrEF: 133 (65) HFpEF: 90 (48) Control: 26 (59)	HFrEF: 21 (11) HFpEF: 84 (41)	NA	22.9 months	Echocardiogram: EAT thickness	HFrEF: 3 (2–6) mm HFpEF: 8 (4–12) mm Control: 5 (3–7) mm
Wang, 2024 (16)	China	Cohort Retro	HFmrEF: 330 HFpEF: 362	Overall: 50 (43–61)	HFmrEF: 57 (51–64) HFpEF: 57 (50–64)	HFmrEF: 27.3 (24.8–30.4) HFpEF: 27.2 (24.1–30.3)	HFmrEF: 201 (61) HFpEF: 205 (57)	NA	HFmrEF: 91 (25) HFpEF: 86 (26)	34 months	CMR: EAT volume	HFmrEF: 67.7 (49.0–86.7) ml/m^2^ HFpEF: 62.7 (45.6–85.6) ml/m^2^
Van woerden, 2022 (17)	Netherlands	Cohort Pro	HFmrEF and HFpEF: 105	Overall: 53 ± 8	Overall: 72 ± 8	Overall: 29.9 ± 5.8	Overall: 52 (50)	NA	Overall: 62 (59)	24 months	CMR: EAT volume	Total EAT: 101.3 ± 29.6 ml/m^2^ atrial EAT: 27.8 ± 14.3 ml/m^2^ ventricular EAT: 74.0 ± 22.2 ml/m^2^
Lin, 2023 (21)	Taiwan	Cohort Pro	HFpEF: 163	HFpEF: 68 ± 9	HFpEF: 61.1 ± 15.3	HFpEF: 25.9 ± 4.2	HFpEF: 103 (63.2)	NA	NA	455 days	CMR: EAT volume	HFpEF: 31 (21–42) g
Lin, 2024 (22)	China	Cohort Retro	HFpEF: 112 Control: 112	NA	HFpEF: 71.9 ± 8.5 Control: 71.9 ± 9.7	NA	HFpEF: 52 (46.5) Control: 52 (46.5)	NA	NA	27 months	CCTA: EAT volume	HFpEF: 56.1 ± 11.9 cm^3^ Control: 38.5 ± 11.1 cm^3^
Nakamori, 2023 (19)	USA	Cohort Retro	HFpEF: 150	HFpEF: 60.1 ± 7.3	HFpEF: 65 ± 12	HFpEF: 30.9 ± 7.9	HFpEF: 82 (55)	HFpEF: 21 (14)	HFpEF: 36 (24)	46months	CMR: EAT volume	HEpEF: 123 ± 47 ml
Jiang, 2024 (23)	China	Cohort Pro	HFpEF: 148 Control: 44	HFpEF: 54 ± 5 Control: 59 ± 7	HFpEF: 72 ± 8 Control: 69 ± 6	HFpEF: 27.4 ± 5.2 Control: 23.7 ± 4.9	HFpEF: 60 (40.5) Control: 23 (47.7)	HFpEF: 21 (14.2)	NA	24months	CMR: EAT volume	HFpEF: 91 (69–112) ml/m^2^ Control: 44 (27–54) ml/m^2^
Liu, 2024 (20)	China	Cohort Retro	Nonischemic HF: 107 Control: 129	NA	Nonischemic HF: 71.8 (9.4) Control: 65.0 (9.3)	Nonischemic HF: 24.89 ± 3.1 Control: 23.8 ± 2.8	Nonischemic HF: 61 (57.0) Control: 78 (60.5)	NA	NA	6 months	CCTA: EAT volume	Nonischemic HF: 55.0 (48.9–64.2) cm^3^ Control: 39.6 (31.3–47.6) cm^3^

Values are mean ± standard deviation, *n* (%), or median [25th quartile–75th quartile].

HF, heart failure; HFrEF, heart failure with reduced ejection fraction; HFpEF, heart failure with peserved ejection fraction; HFmrEF, heart failure with mid-range ejection fraction; LVEF, left ventricular ejection fraction; AF, atrial fibrillation; BMI, body mass index; EAT, epicardial adipose tissue; CMR, cardiac magnetic resonence; CCTA, coronary computed tomography angiography; NA, not significant; Pro, prospective; Retro, retrospective.

### 3.3 Relationship between EAT and AEs

**Figure 2:**
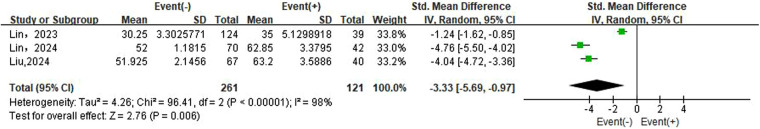
The forest plot of the SMD in baseline EAT and the 95% CI between the two groups described above.

Three studies were included investigating differences in EAT in HF people without and with AEs. EAT volume was higher in HF patients with AEs than those who did not experience AEs (Table 2). The pooled results showed a difference of 3.33 (95% CI: 0.96–5.69, *p* = 0.006, *I*^2^ = 98%) between the two groups (Figure 2).

**Table 2:** Baseline comparison of EAT between HF patients with and without AEs.

Author, year	Study population	Event		No event	
		Patient (N1)	EAT measurement	Patient (N2)	EAT measurement
Lin, 2024	HFpEF: 112	42	63.3 (55.8,69) cm^3^	70	52.4 (48.8,54.4) cm^3^
Liu, 2024	Nonischemic HF: 107	40	63.9 (55.5,69.5) cm^3^	67	51.7 (47.1,57.2) cm^3^
Lin, 2023	HFpEF: 163	39	35 (25,45) g	124	31 (21,38) g

### 3.4 The predictive value of EAT for adverse outcomes

**Figure 3:**
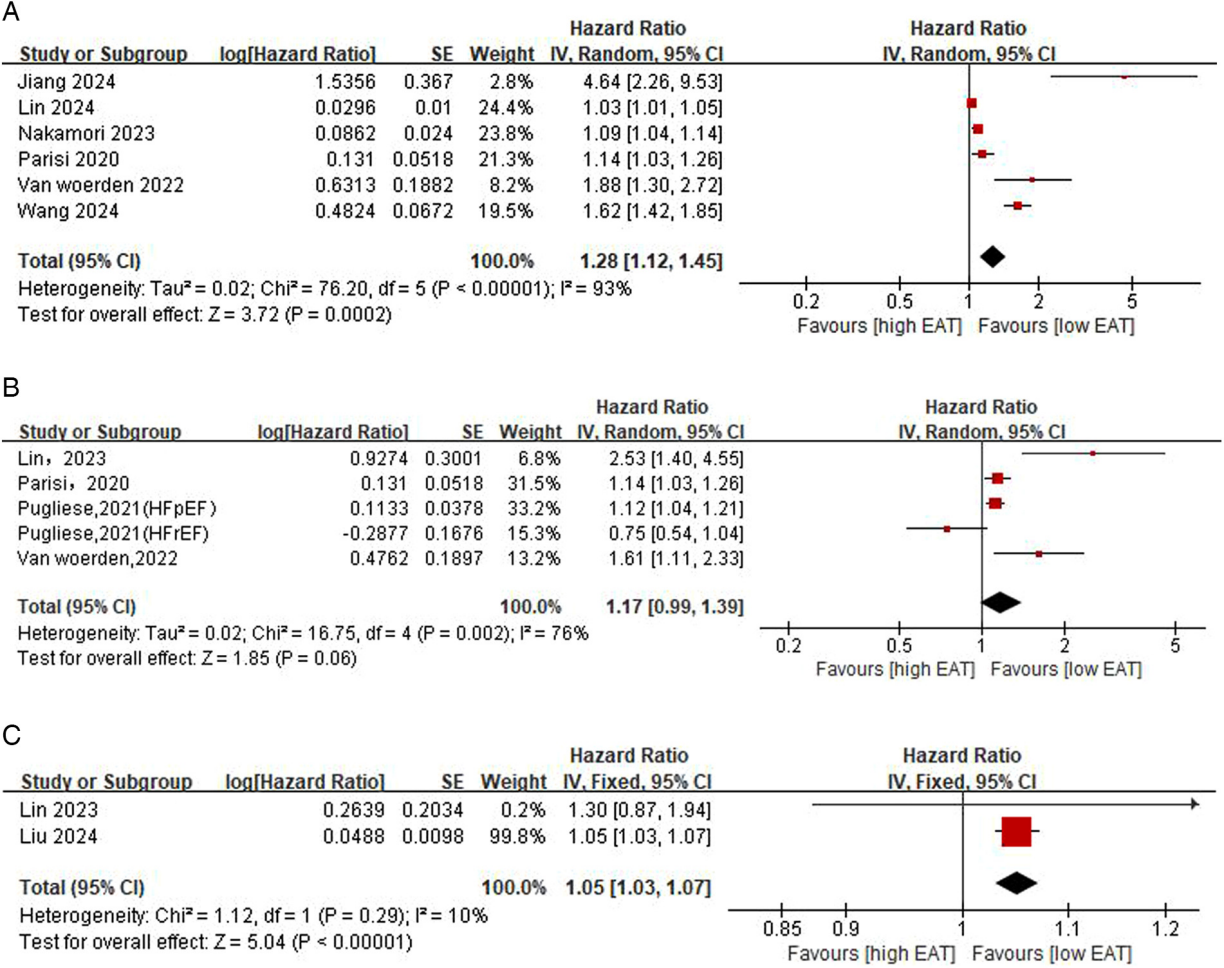
Epicardial adipose tissue (EAT; per 1-unit increase) predicts the pooled HR for the primary outcome [**(A)** composite outcome of ACM and HF readmission] and the secondary outcome [**(B)** composite outcome of cardiovascular death and HF readmission **(C)** HF readmission].

Figure 3A shows the correlation between EAT and primary outcomes. According to results from six studies, the risk of the primary outcome presented a 28% increase in statistical level for each additional unit of EAT (HR: 1.28, 95% CI: 1.12–1.45, *p* = 0.0002, *I*^2^ = 93%). In subgroup analyses based on LVEF levels, the risk of the primary outcome was significantly increased for each unit increase in EAT in the EF ≥50% patient group (HR: 1.20, 95% CI: 1.06–1.37, *p* = 0.006, *I*^2^ = 92%), whereas the pooled HR of EAT was not statistically significant in the EF <50% patient group (HR: 1.38, 95% CI: 0.94–2.02, *p* = 0.10, *I*^2^ = 92%) (Supplementary Figure S1). In subgroup analyses based on EAT indicators, EAT thickness and EAT volume were both significantly associated with major prognosis, but only in HF patients with EF ≤40% and EF >40%, respectively (Supplementary Figure S2). In addition, in subgroup analyses based on different countries and ages, there was a significant increase in pooled HR in all patient populations except patients aged ≥70 (Supplementary Figures S3, S4).

Figures 3B,C show the correlation between EAT and secondary outcomes. The pooled results from two studies showed that increased EAT indicated a higher risk of HF rehospitalization (HR: 1.05, CI: 1.03–1.07, *p* < 0.001, *I*^2^ = 10%). Specific to cardiovascular death and HF hospitalization, the results of four studies demonstrated that increased EAT was accompanied by an elevated risk of composite outcomes (HR: 1.17, CI: 0.99–1.39, *P* = 0.06, *I*^2^ = 76%), but this was not statistically significant. In subgroup analyses based on HF levels, the results showed that EAT in HF patients with EF >40% and EF ≤40% did not exhibit an obvious relevance to the risk of the composite outcome of cardiovascular death and HF hospitalization (Supplementary Figure S5).

**Figure 4:**
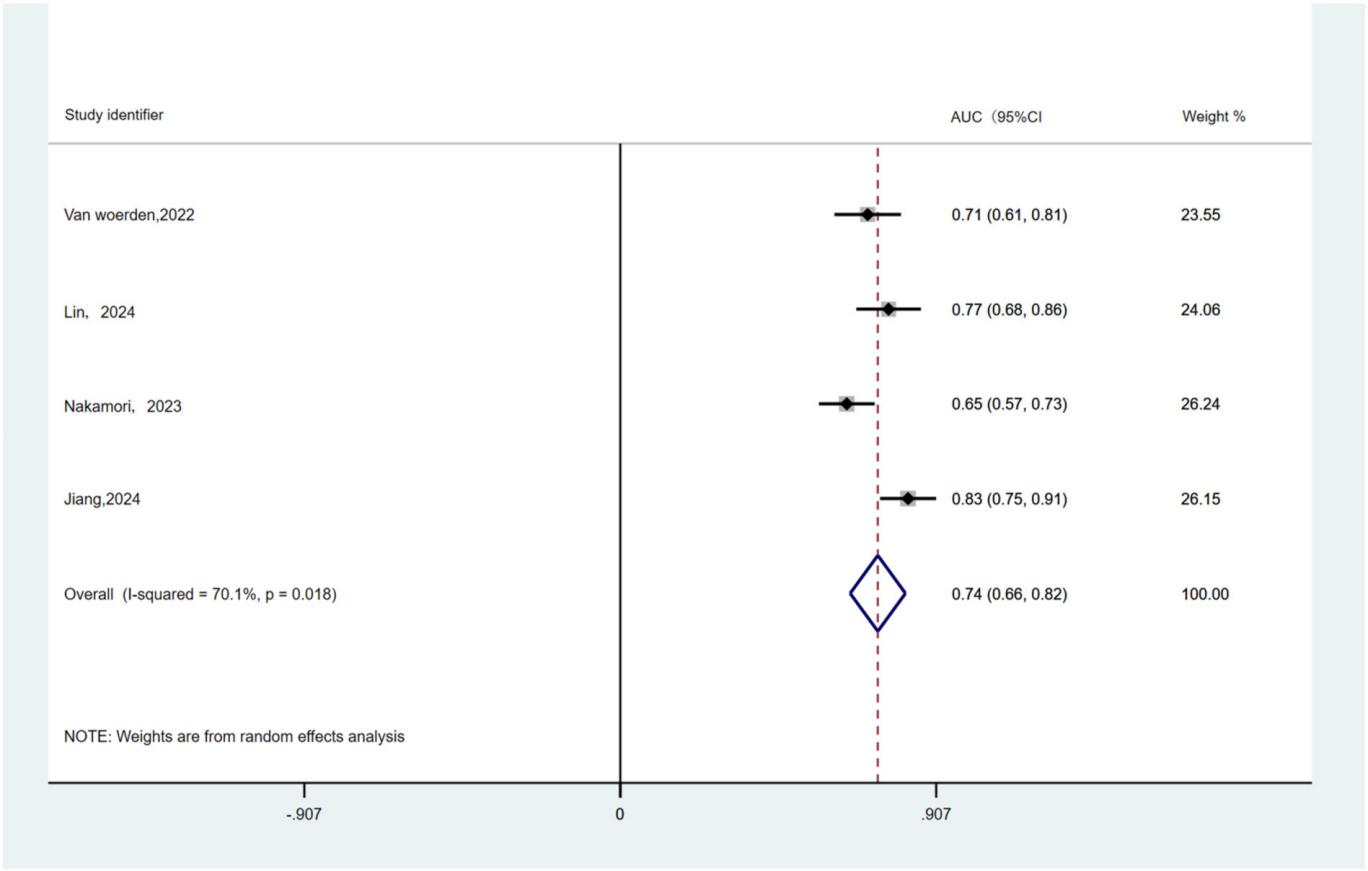
Pooled AUC values for EAT used to predict primary outcomes.

Table 3 summarizes the cut-off and AUC values used by EAT to predict primary outcomes. Four studies involving HF patients with EF >40% were included predominantly, with AUC values ranging from 0.65 to 0.83 for EAT volume to predict the primary outcome. AUC results were pooled by a random-effects model (Figure 4). The combined AUC value for predicting risk of primary outcome using EAT volume was 0.74 (95% CI: 0.66–0.82, *p* = 0.018, *I*^2^ = 70.1%).

**Table 3:** Cut-offs and AUC for EAT to identify risk stratification for primary outcomes.

Author, year	Study population	EAT measurement	main outcome	EAT threshold	ROC-AUC
Van woerden, 2022	HFmrEF and HFpEF: 105	CMR: EAT volume	Composite of all-cause mortality and first HF hospitalizations	106 ml/m^2^	0.71
Lin, 2024	HFpEF: 112	CCTA: EAT volume	Composite endpoint of all-cause death or heart failure readmission	56.29 cm^3^	0.77
Nakamori, 2023	HFpEF: 150	CMR: EAT volume	Composite outcome of all-cause mortality and first HF hospitalizations	130 ml	0.65 (0.56–0.72)
Jiang, 2024	HFpEF: 148	CMR: EAT volume	Composite of all-cause mortality and rehospitalization for Heart failure, myocardial infarction or stroke	91.5 ml/m^2^	0.826 (0.755–0.897)

### 3.5 Sensitivity analysis

The sensitivity analyses were performed on the findings of EAT with respect to the primary outcome (Supplementary Figure S5). The results showed that after the exclusion of the Lin and Nakamori studies, respectively, there was a large change in the pooled HR of the remaining studies, indicating that these two studies more greatly affected the overall pooled results, which may be due to the fact that these two studies were given more weight. Nonetheless, there was no statistically significant change in the correlation between EAT and primary outcome. Therefore, the results of the pooled effect of EAT and primary outcomes should be robust and reliable.

### 3.6 Publication bias

According to the funnel plot and Egger test (*P* = 0.008), there may have been publication bias in six studies reporting HR between EAT and the primary outcome, but the Begg test did not report publication bias (*P* = 0.06) (Supplementary Figure S7). For this reason, we conducted a trim and fill analysis, which showed a total of 2 missing studies, but the pooled results before and after filling were stable, so publication bias did not have a significant effect on the overall estimates (Supplementary Figure S8). In addition, the funnel plot after the filling of the missing study showed a symmetrical distribution up and down (Supplementary Figure S9).

## 4 Discussion

In our systematic review and meta-analysis of nine cohort studies involving 1,939 HF patients and 329 control populations, We analyzed HR data between EAT and adverse outcomes in HF patients. The primary findings indicate that in the analysis of the primary outcome, an increase of EAT exhibited an significant relevance to the primary outcomes in HF patients. In subgroup analyses, EAT was still related to the risk of the primary outcome in patients with EF ≥50%, while did not exhibit any relevance to that in patients with EF <50%. And the AUC value for predicting the primary outcome based on EAT was high in patients with EF >40%. In the analysis of secondary outcomes, EAT presented no correlation with the risk of the composite outcome of cardiovascular death and hospitalization for HF patients with EF >40% and EF ≤40%, while increased EAT in HF patients indicated a higher possibility of readmission for HF.

EAT has a protective brown fat profile and is able to act as a metabolic reservoir for HFrEF (24). In times of high metabolic demand, such as cardiac ischemia, EAT can help improve cardiac function and contractility through free fatty acids as local energy sources (25). And the pathophysiological pathways associated with increased metabolism in patients with HFrEF are upregulated (26). Thus, the thinner EAT may reflect the potential increased energy demand of the heart in patients with HFrEF. Lower EAT in HFrEF patients also correlates significantly with poorer systolic function, myocardial damage, and cardiac remodeling (27). In HFpEF, significant EAT accumulation may lead to myocardial steatosis or adipose tissue infiltration of adjacent myocardium (28), and secretion of a wide range of pathopathological, pro-inflammatory, and atherosclerotic adipogenic factors through paracrine, endocrine, and vascular pathways (29), thereby contributing to hemodynamic derangement (30–32), myocardial fibrosis, and sclerosis (29), leading to cardiac impairment and poor prognosis (9, 33). In addition, large accumulations of EAT may also exert mechanical compression on the heart, thereby limiting encapsulated myocardial expansion, which can lead to diastolic dysfunction and increase cardiac filling pressures (30, 34). Although the mechanism between EAT and impaired prognosis are not fully understood in HFrEF and HFpEF patients, the role of EAT in the pathophysiologic process of HF shows the possibility that quantitative detection of EAT may independently predict HF patients' poor prognosis in clinical practice. In fact, EAT is related to prognosis in patients suffering from coronary artery disease and type 2 diabetes as well as in individuals who do not have cardiovascular disease (35–37).

At present, the prognostic assessment of HF is mainly based on independent and routine risk factors such as demographic characteristics, biomarkers, and imaging parameters. Kenneth et al. (38) used age, New York Heart Association functional class, atrial fibrillation, chronic obstructive pulmonary disease, chronic kidney disease, LVEF, and diabetes mellitus to predict all-cause mortality in HFrEF, and the C-statistics of this model were 0.75 and 0.74 in the development and validation cohorts, respectively. Komajda et al. (39) showed that N-terminal pro-B-type natriuretic peptide, age, diabetes mellitus, and LVEF were the strongest independent factors for all-cause mortality in HFpEF, with a C-statistic of 0.736 for the constructed model. It can be seen that models constructed by these factors have only moderate clinical performance in predicting AEs and that different types of HF may require different clinical predictors to predict prognosis. Our meta-analysis indicated that EAT accumulation could trigger a higher risk of the primary outcome in HF patients. And in the analysis of EAT and HF readmission events, although the results of Liu et al. (20) and Lin et al. (21) were inconsistent, our results found an intense relevance of EAT to HF readmission risk. In line with this, a prospective cohort study showed that pericardial fat hinted a higher risk in HF patients, particularly HFpEF patients (35). This suggests that EAT may be able to be used as a useful parameter for assessing the clinical prognosis of HF, but the predictive value of EAT for poor prognosis in the entire heart failure cohort is not known. In HF patients with EF ≥50%, EAT similarly demonstrated an association with a higher risk of the primary outcome and showed higher pooled AUC values in predicting outcomes. Additionaly, Wang et al. (16) engaged in further validating the incremental predictive value of EAT for baseline model performance by adding it to the baseline multivariate model. The results show that EAT can significantly improve the performance of predicting the primary outcome (net weight classification index improvement by 8.4%). Thus, EAT holds promise as a clinical predictor for HF patients with EF ≥50%, contributing to the development of more effective predictive models that can significantly improve risk prediction.

However, the association between EAT and the primary outcome does not exist for those with EF <50%. This may be due to the fact that the positive effects of EAT thickening on ventricular structure and function are counterbalanced by an increased propensity for ventricular arrhythmias (40). Inconsistent with our findings, Paris et al. (18) included 69 systolic HF patients who received ICD implantation, and showed that an increase of EAT thickness was associated with HF hospitalization risk and HF-related mortality. Pugliese et al. (13) argued that a decrease in EAT thickness was significantly associated with an adverse prognosis in HFrEF patients. All these findings differ from the result of our study, which may be due to the inconsistency of the included patient populations or the inclusion of both HFrEF and HFmrEF patients in our pooled results. Thus, the predictive role of EAT for main outcome in HF patients with EF <50% is debated and further clinical studies are still needed. In addition, This meta-analysis showed no significant correlation between EAT and the composite outcome of cardiovascular death and HF hospitalization, no matter HFrEF or HFpEF patients. In contrast, Both Van woerden et al. (17) and Lin et al. (21) showed a correlation between increased EAT and cardiovascular death and HF hospitalization in HFpEF. However, according to a correlation analysis of cardiovascular mortality outcomes in the study by Lin et al. (21), EAT did not relate to the patient's risk. Therefore, the relevance of EAT to the composite outcomes of cardiovascular death and HF hospitalization needs to be further studied in HFrEF and HFpEF.

Notably, the included studies in the analysis had a high degree of heterogeneity, which may be due to differences in study methodology, population characteristics, follow-up times, and outcome definitions and EAT measurement protocols. EAT may have prognostic differences across geographic regions and imaging modalities. All studies included were from patient populations in different countries and regions, and the patient populations studied also included different HF types. The study methodology was also inconsistent, containing prospective and retrospective studies. Most importantly, differences in EAT measurement protocols may be the main cause of heterogeneity. Some of the included studies used echocardiography to measure EAT thickness, while others used CMR and CCTA to measure EAT volume. And one study calculated the mean thickness over 3 cardiac cycles, while the other one considered 2 cardiac cycles. Some studies used a 1.5T CMR scanner to delineate and measure EAT volumes from the short-axis view, while others used 3T from the short-axis four-chamber view and measured EAT volumes in different units of measure, including ml, cm^3^, ml/cm^2^. This observation highlights the lack of standardization of measurement techniques and EAT metrics, which challenges the application of EAT as a risk stratification tool. Thus, future studies require standardized measurement techniques and defined quantitative metrics and thresholds for EAT to enable accurate risk stratification.

### 4.1 Limitations

First, There was substantial heterogeneity among the studies included in this meta-analysis. Although this study corrected for these differences using a random effects model and conducted subgroup and sensitivity analyses, the results need to be interpreted with caution. Second, publication bias was found using the funnel plot and Egger's test, but this study went on to perform a cut-and-fill analysis and confirmed that publication bias had a small effect on the study results. Third, in our meta-analysis, there were fewer studies that included EAT in relation to the prognosis of HFrEF, so the prognostic value of EAT in HFrEF was unclear. Fourth, of note, echocardiography can be used to measure EAT thickness, whereas CMR and CCTA are able to measure EAT volume. But due to the paucity of studies on the prognostic relevance of EAT derived from CCTA and echocardiography included in this study, it is difficult to stratify the assessment of the relationship between EAT and prognosis in HF based on imaging modality. Finally, due to the lack of harmonized standards and metrics for EAT measurement, we are unable to determine the cut-off value of EAT and its diagnostic accuracy on the ROC curve. Therefore, it is necessary to obtain standardized measurement protocols and Improved EAT quantification threshold.

## 5 Conclusion

The systematic review and meta-analysis here indicated the significant association of EAT to the risk of the composite outcome of the primary outcome and HF readmission in people with HF, but not to cardiovascular mortality and HF hospitalization. In addition, although the relevance of EAT to cardiovascular death and HF hospitalization is controversial in patients with HFpEF, their EAT triggers a higher risk of the primary outcome. The association of EAT with ACM, cardiovascular death, HF hospitalization, and their composite outcomes in patients with HFrEF still needs to be confirmed by further research. But It is important to note that there was high heterogeneity among the included studies, due to different EAT measurement protocols, a further step is needed to identify standardized measurement protocols and defined measurement units.

## Data Availability

The original contributions presented in the study are included in the article/Supplementary Material, further inquiries can be directed to the corresponding authors.
