# Supplementary material for: Prognostic value of epicardial adipose tissue in heart failure: a systematic review and meta-analysis
Source: Front Cardiovasc Med. 2025 Jun 26;12:1618614. doi: 10.3389/fcvm.2025.1618614 (PMC12240945; doi:10.3389/fcvm.2025.1618614)
Supplement: Supplementary file 1 [file Datasheet1.pdf]

Table S1 Methodological quality assessment using the Newcastle-Ottawa Scale for included studies

Publication	Selection				Comparability	Outcome			Total score
	S1	S2	S3	S4	C	O1	O2	O3	
Parisi， 2020	★	★	★	★	★—	★	★	★	8
Puglì ese, 2021	★	★	★	★	★★	★	★	★	9
Wang, 2024	★	★	★	★	★★	★	★	★	9
Van woerden, 2022	★	★	★	★	★★	★	★	★	9
Lin， 2023	★	★	★	★	★★	★	★	★	9
Lin， 2024	★	★	★	★	★★	★	★	—	8
Nakamori， 2023	★	★	★	★	★—	—	★	★	7
Jiang, 2024	★	★	★	★	★—	★	★	★	8
Liu, 2024	★	★	★	★	★★	★	—	—	7

S1, Representative-ness; S2, Selection of non-exposed; S3, Ascertainment of exposure S4, Outcome not present at start; C, Comparability on most important factors and other risk factors; O1, Assessment of outcome; O2, Long enough follow-up (median⩾1 year); O3, the adequacy the follow-up. the higher the score the higher the quality of the study, 0 ~4 was categorized as low quality study, 5-6 as moderate quality study, and 7-9 as high quality study.

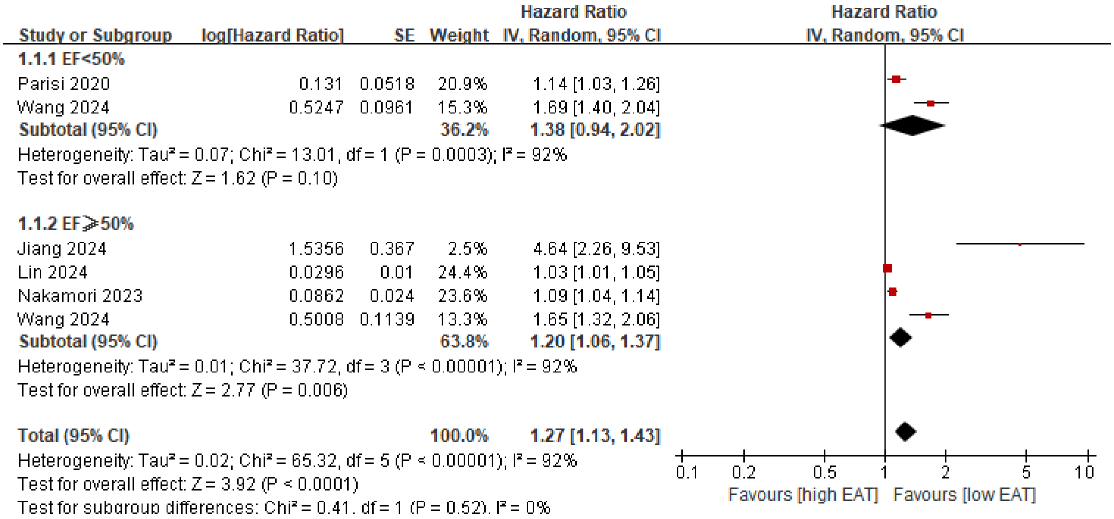

Figure S1 Subgroup analyses based on LVEF phenotypes showed that the pooled HR of EAT (per 1-unit increase) for predicting the primary outcome

Study or Subgroup	log[Hazard Ratio]	SE	Weight	Hazard Ratio IV, Random, 95% CI
**1.2.1 EAT thickness**				
Parisi 2020	0.131	0.0518	21.3%	1.14 [1.03, 1.26]
**Subtotal (95% CI)**			**21.3%**	**1.14 [1.03, 1.26]**
Heterogeneity: Not applicable				
Test for overall effect: Z = 2.53 (P = 0.01)				
**1.2.2 EAT volume**				
Jiang 2024	1.5356	0.367	2.8%	4.64 [2.26, 9.53]
Lin 2024	0.0296	0.01	24.4%	1.03 [1.01, 1.05]
Nakamori 2023	0.0862	0.024	23.8%	1.09 [1.04, 1.14]
Van woerden 2022	0.6313	0.1882	8.2%	1.88 [1.30, 2.72]
Wang 2024	0.4824	0.0672	19.5%	1.62 [1.42, 1.85]
**Subtotal (95% CI)**			**78.7%**	**1.33 [1.14, 1.56]**
Heterogeneity: Tau² = 0.02; Chi² = 73.74, df = 4 (P < 0.00001); I² = 95%				
Test for overall effect: Z = 3.63 (P = 0.0003)				
**Total (95% CI)**			**100.0%**	**1.28 [1.12, 1.45]**
Heterogeneity: Tau² = 0.02; Chi² = 76.20, df = 5 (P < 0.00001); I² = 93%				
Test for overall effect: Z = 3.72 (P = 0.0002)				
Test for subgroup differences: Chi² = 2.73, df = 1 (P = 0.10), I² = 63.3%				

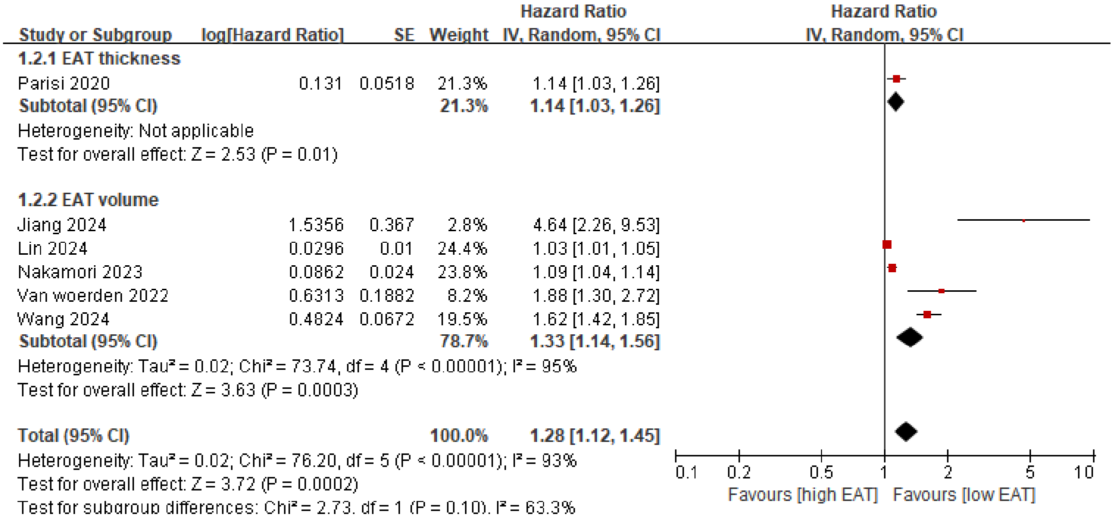

Figure S2 Subgroup analyses based on EAT indicators showed that the pooled HR of EAT (per 1-unit increase) for predicting the primary outcome

Study or Subgroup	log[Hazard Ratio]	SE	Weight	Hazard Ratio IV, Random, 95% CI
**1.3.1 china**				
Jiang 2024	1.5356	0.367	2.8%	4.64 [2.26, 9.53]
Lin 2024	0.0296	0.01	24.4%	1.03 [1.01, 1.05]
Wang 2024	0.4824	0.0672	19.5%	1.62 [1.42, 1.85]
**Subtotal (95% CI)**			**46.8%**	**1.67 [1.06, 2.62]**
Heterogeneity: Tau² = 0.13; Chi² = 61.03, df = 2 (P < 0.00001); I² = 97%				
Test for overall effect: Z = 2.20 (P = 0.03)				
**1.3.2 other country**				
Nakamori 2023	0.0862	0.024	23.8%	1.09 [1.04, 1.14]
Parisi 2020	0.131	0.0518	21.3%	1.14 [1.03, 1.26]
Van woerden 2022	0.6313	0.1882	8.2%	1.88 [1.30, 2.72]
**Subtotal (95% CI)**			**53.2%**	**1.18 [1.03, 1.36]**
Heterogeneity: Tau² = 0.01; Chi² = 8.66, df = 2 (P = 0.01); I² = 77%				
Test for overall effect: Z = 2.31 (P = 0.02)				
**Total (95% CI)**			**100.0%**	**1.28 [1.12, 1.45]**
Heterogeneity: Tau² = 0.02; Chi² = 76.20, df = 5 (P < 0.00001); I² = 93%				
Test for overall effect: Z = 3.72 (P = 0.0002)				
Test for subgroup differences: Chi² = 2.02, df = 1 (P = 0.16), I² = 50.5%				

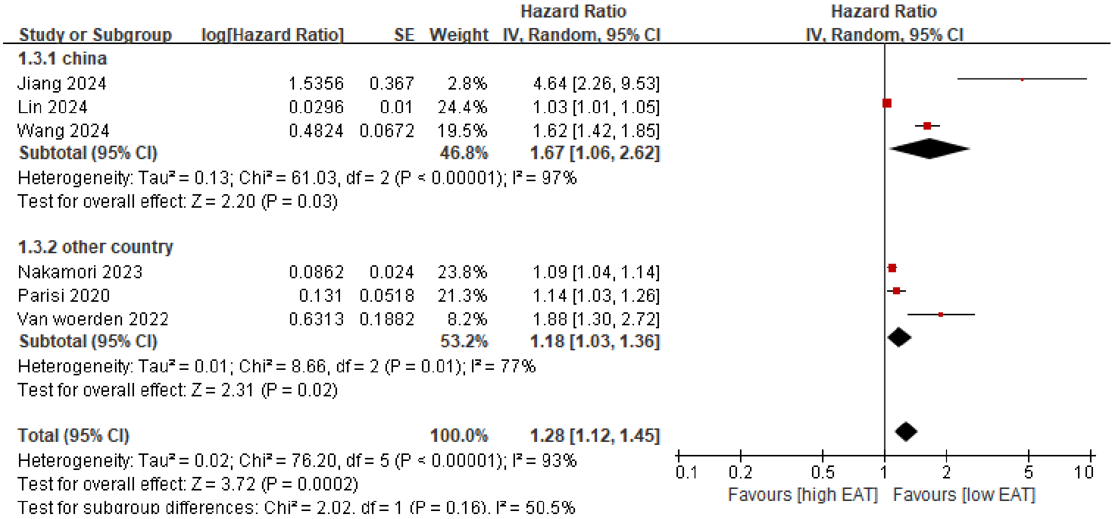

Figure S3 Subgroup analyses based on different country showed that the pooled HR of EAT (per 1-unit increase) for predicting the primary outcome

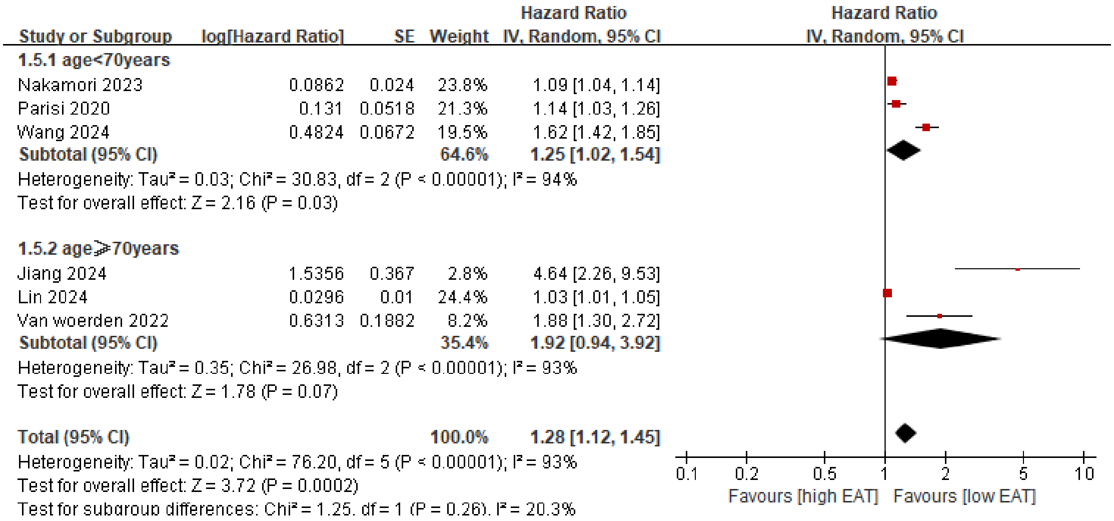

Figure S4 Subgroup analyses based on population age showed that the pooled HR of EAT (per 1-unit increase) for predicting the primary outcome

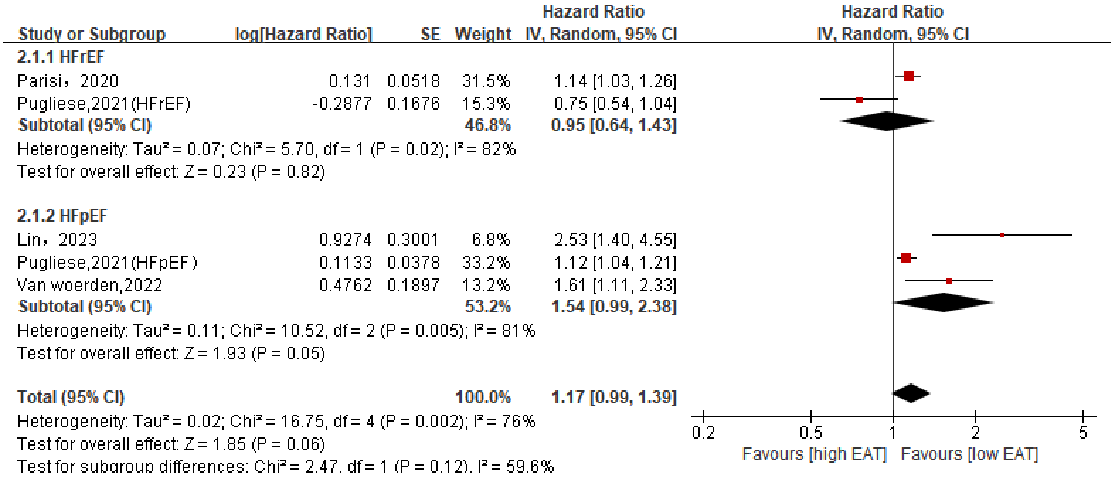

Figure S5 Subgroup analyses based on LVEF phenotypes showed that the pooled HR of EAT (per 1-unit increase) for predicting the secondary outcome

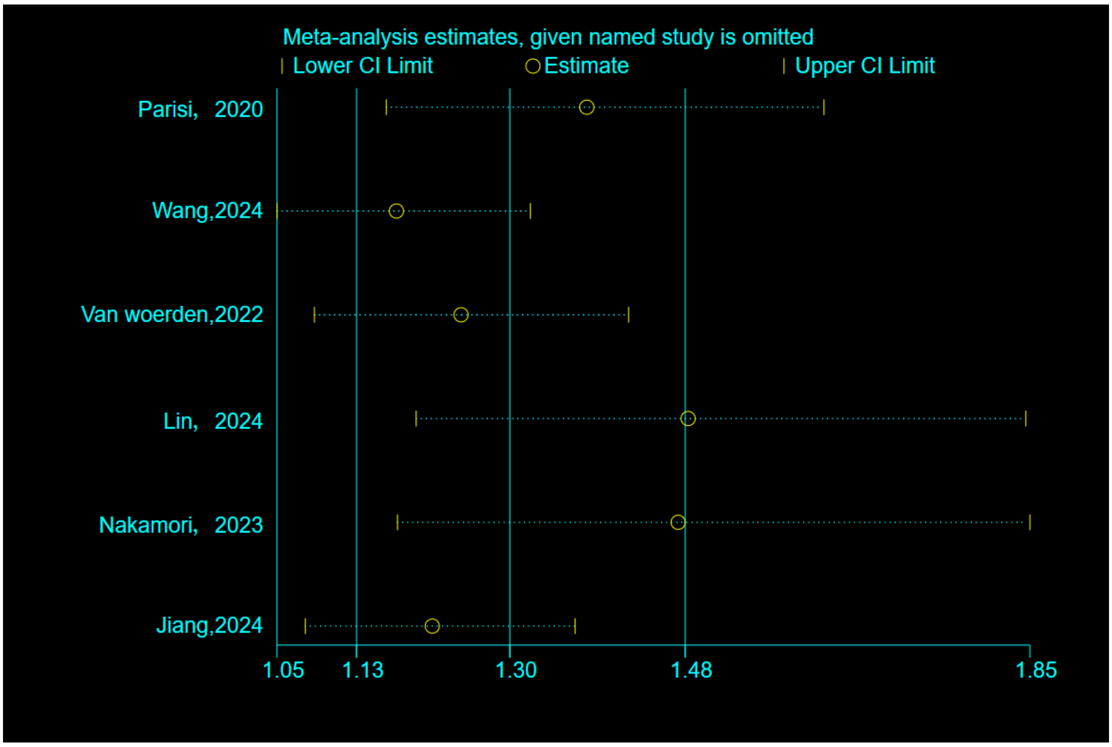

Figure S6 Sensitivity analysis of EAT to the pooled results of the primary outcome by excluding individual studies one by one

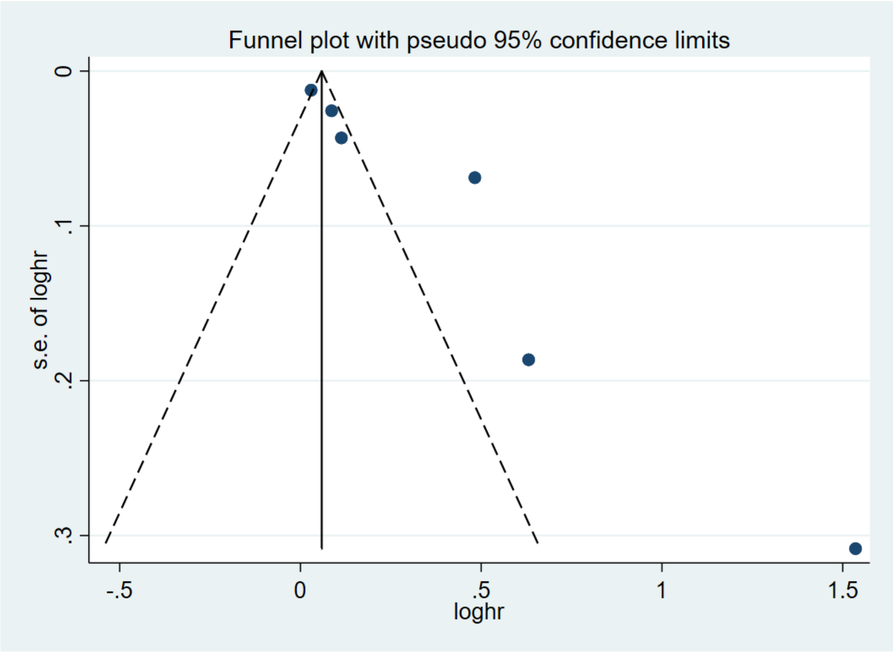

```
Begg's Test

  adj. Kendall's Score (P-Q) =       11
          Std. Dev. of Score =     5.32
           Number of Studies =        6
                          z  =     2.07
                  Pr > |z|  =    0.039
                          z  =     1.88  (continuity corrected)
                  Pr > |z|  =    0.060  (continuity corrected)
```

Egger's test

Std_Eff	Coef.	Std. Err.	t	P>|t|	[95% Conf.	Interval]
slope	-.031912	.0252269	-1.27	0.275	-.101953	.038129
bias	4.843771	.9709495	4.99	0.008	2.147983	7.539559

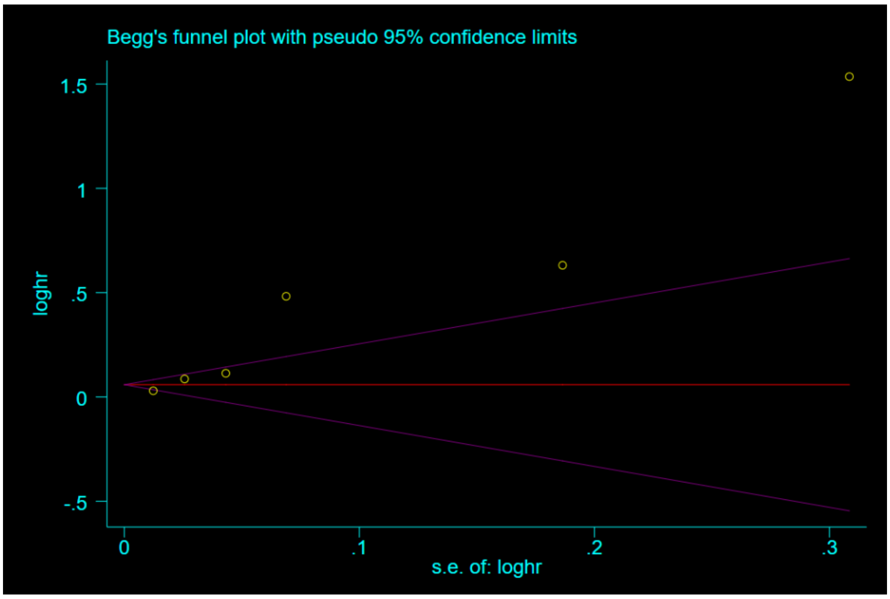

Figure S7 Three methods，Funnel plots，Egger's test and Begg's test，were used to assess publication bias in the six studies that reported HR between EAT and primary outcomes

```
Meta-analysis

       |  Pooled      95% CI         Asymptotic      No. of
Method |     Est   Lower   Upper  z_value  p_value   studies
-------+----------------------------------------------------
Fixed  |   0.059   0.038   0.080   5.541    0.000      6
Random |   0.261   0.126   0.395   3.804    0.000

Test for heterogeneity: Q= 78.561 on 5 degrees of freedom (p= 0.000)
Moment-based estimate of between studies variance =  0.020

Trimming estimator: Linear
Meta-analysis type: Random-effects model

iteration |  estimate    Tn    # to trim     diff
----------+--------------------------------------
    1     |    0.261     14        1          21
    2     |    0.195     15        2           2
    3     |    0.157     15        2           0

Filled
Meta-analysis (exponential form)

       |  Pooled      95% CI         Asymptotic      No. of
Method |     Est   Lower   Upper  z_value  p_value   studies
-------+----------------------------------------------------
Fixed  |   1.058   1.036   1.080   5.296    0.000      8
Random |   1.179   1.027   1.353   2.341    0.019

Test for heterogeneity: Q= 99.751 on 7 degrees of freedom (p= 0.000)
Moment-based estimate of between studies variance =  0.025
```

Figure S8 The trim and fill analysis were used to assess the effect of publication bias on pooled results

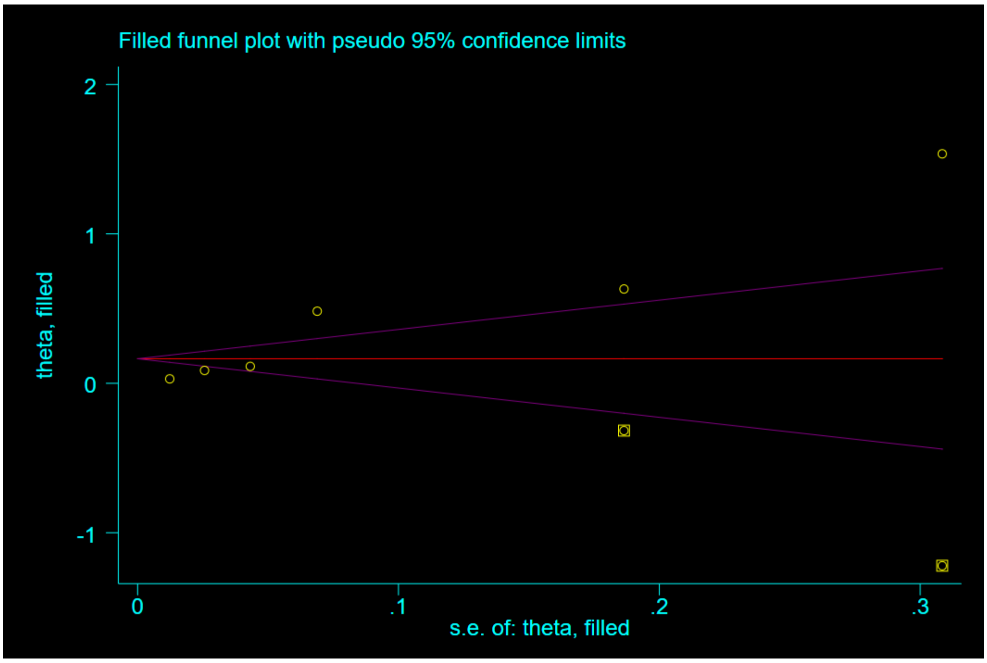

Figure S9 Using funnel plots to assess publication bias after filling in missing studies
